# The status quo of short videos as a health information source of *Helicobacter pylori*: a cross-sectional study

**DOI:** 10.3389/fpubh.2023.1344212

**Published:** 2024-01-08

**Authors:** Yongkang Lai, Foqiang Liao, Zixuan He, Weiguo Lai, Chunping Zhu, Yiqi Du, Zhaoshen Li

**Affiliations:** ^1^Department of Gastroenterology, Ganzhou People’s Hospital, Jiangxi Medical College, Nanchang University, Ganzhou, China; ^2^Department of Gastroenterology, Shanghai Changhai Hospital, Naval Medical University, Shanghai, China; ^3^Department of Gastroenterology, The First Affiliated Hospital of Nanchang University, Nanchang, China

**Keywords:** *Helicobacter pylori*, short video, health information, quality, reliability

## Abstract

**Background:**

Health education about *Helicobacter pylori (H. pylori)* is one of the most effective methods to prevent *H. pylori* infection and standardize *H. pylori* eradication treatment. Short videos enable people to absorb and remember information more easily and are an important source of health education. This study aimed to assess the information quality of *H. pylori*-related videos on Chinese short video-sharing platforms.

**Methods:**

A total of 242 *H. pylori*-related videos from three Chinese short video-sharing platforms with the most users, TikTok, Bilibili, and Kwai, were retrieved. The Global Quality Score (GQS) and the modified DISCERN tool were used to assess the quality and content of videos, respectively. Additionally, comparative analyzes of videos based on different sources and common *H. pylori* issues were also conducted.

**Results:**

The median GQS score and DISCERN score was 2 for *H. pylori*-related videos analyzed in this study. Non-gastroenterologists posted the most *H. pylori-*related videos (136/242, 56.2%). Videos from gastroenterologists (51/242, 21.0%) had the highest GQS and DISCERN scores, with a median of 3. Few videos had content on family-based *H. pylori* infection control and management (5.8%), whether all *H. pylori*-positive patients need to undergo eradication treatment (27.7%), and the adverse effects of *H. pylori* eradication therapy (16.1%).

**Conclusion:**

Generally, the content and quality of the information in *H. pylori*-related videos were unsatisfactory, and the quality of the video correlated with the source of the video. Videos from gastroenterologists provided more correct guidance with higher-quality information on the prevention and treatment of *H. pylori* infection.

## Highlights

- **What is already known on this topic**—*H. pylori* infection is the main cause of gastric cancer, but most people do not know about *H. pylori*. The short video platform is favored by the majority of users because it can spread health information through vivid graphics and videos.
- **What this study adds**—This study evaluated the information quality of *H. pylori*-related videos on short video platforms in China, hoping to provide some suggestions for improving short video content on *H. pylori*.
- **How this study might affect research, practice or policy**—Overall, the content and quality of the information in *H. pylori*-related videos were unsatisfactory, and the quality of the video correlated with the source of the video. Short video platforms should be encouraged to strengthen management to prevent the spread of misinformation through the Internet. In addition, more professionals should be advocated to release authoritative videos.

## Introduction

*Helicobacter pylori (H. pylori)* is a microaerophilic gram-negative helical-shaped bacterium that has infected almost half of the world population (1). Usually transmitted during childhood through oral-oral or fecal-oral routes, *H. pylori* secretes a series of virulence factors after colonizing the stomach epithelium and may lead to a range of gastrointestinal and extra-gastrointestinal diseases, including chronic gastritis, peptic ulcers, gastric adenocarcinoma, iron-deficiency anemia, and mucosa-associated lymphoid tissue lymphoma, which cause heavy economic and healthcare burden (2–5). Early in 1994, *H. pylori* was recognized as a Class I (highest class) carcinogen for gastric cancer by the World Health Organization (WHO), and in 2014, WHO called for the worldwide eradication of *H. pylori* as an important strategy for preventing gastric cancer (6). The 15th edition of the US Report on Carcinogens published in 2021 classified *H. pylori* as a definite carcinogen (7). Therefore, eradicating *H. pylori* is of great importance for maintaining human health and reducing economic burden.

There is growing public awareness that *H. pylori* infection is the primary cause of gastric cancer and curing the infection can reduce the incidence or prevent the development of gastric cancer (8). However, there is a lack of knowledge on how to prevent *H. pylori* infection and standardize the eradication treatment of *H. pylori*. Additionally, there is also a certain level of fear and anxiety about *H. pylori* among people. There are concerns about how to detect *H. pylori*, which method is the gold standard for *H. pylori* eradication, and whether all positive tests indicate *H. pylori* infection. Furthermore, the treatment of *H. pylori* infection requires the long-term use of two antibiotics in large quantities. However, irregular medication, lack of knowledge about the adverse effects of treatment regimens, and unauthorized discontinuation of drugs lead to eradication failure. Moreover, whether *H. pylori* eradication is required for all age groups and whether eradication should be done at the family level impact the success or failure of *H. pylori* eradication. Therefore, understanding *H. pylori* and standardized treatment of *H. pylori* infection are important to increase the *H. pylori* eradication rate.

Emerging technologies are developing improved methods of health communication between doctors and patients, empowering patients to get disease-related information (9). Short video platforms, such as TikTok, Kwai, and Bilibili, are replacing traditional textual information formats with more acceptable graphic video information allowing patients to absorb and remember information more easily (10–12). According to recent evidence, active use of social media to obtain disease-related information is associated with a good prognosis for patients, which empowers patients regarding self-disease management and helps to reduce the financial burden of health care (13, 14). Besides, with the increasing number of short video users in China, 934 million by 2021, short videos are certainly an efficient format to standardize testing and treatment of *H. pylori* in the population (14).

However, despite the promising potential of short videos, there are certain accompanying problems that cannot be ignored. Among them, the information quality of short videos deserves our attention the most. The abundance of short videos has increased the possibility of faulty health information, which may lead patients to make incorrect judgments about disease management (15, 16). Moreover, low-quality video health content can put patients through a more convoluted disease treatment process and can add more financial burden to patients (17). Therefore, examining the quality of short videos as a health information source and providing patients guidance about the standardized treatment of *H. pylori* infection are important for *H. pylori* eradication.

The quality of *H. pylori* information on video-based social media platforms has not been fully assessed. This study aims to examine the information quality of *H. pylori* videos on short video platforms in China, hoping to provide some suggestions for improving short video content on *H. pylori*.

## Methods

### Search strategy and data processing

This was an observational retrospective study based on videos collected from TikTok, Bilibili, and Kwai, the three most popular short video platforms in China. Using search keywords “幽门螺杆菌” or “幽门螺旋杆菌” (“*H. pylori*” in Chinese), this study retrieved the first 100 Chinese language videos each from TikTok, Bilibili, and Kwai. The entire search process was conducted and completed during March 10–15, 2023. The exclusion criteria were as follows: (1) videos with duplicate content; (2) videos with incomplete data; (3) videos with no sound/poor sound quality; (4) videos irrelevant to the topic. Videos for advertisement or commercial purposes were also excluded. The videos were independently reviewed and categorized by two qualified physicians (Lai YK and Liao FQ), who worked in the department of gastroenterology in a tertiary hospital and had extensive experience in managing patients with *H. pylori* infection, and discrepancies, if any, were resolved by consensus.

Basic information, including publication date, name and type of the uploader, physician title and department, video duration (seconds), and the number of likes, favorites, and shares, was extracted from each video and recorded in Excel (Microsoft Corporation). Patients or the public WERE NOT involved in the design, or conduct, or reporting, or dissemination plans of our research.

### Evaluation methodologies and procedure

The content and information quality of the videos were evaluated using the modified DISCERN tool and the Global Quality Score (GQS), respectively. DISCERN, first proposed by Goobie et al., is a widely validated and applied tool to help consumers and health professionals assess the quality of health-related content in videos (10, 18, 19). The tool assesses videos primarily through five questions (see Supplementary Table S1), which are scored either “1” or “0” according to whether the answer is “yes” or “no,” with a minimum score of 0 out of 5. The GQS is another widely used tool for assessing the quality of health information in videos, with response selection based on a 5-point scale from 1 (poor quality) to 5 (good quality) (10, 20, 21). Details of this tool are presented in Supplementary Table S2. In addition, we used another video scoring tool from Goobie et al. that scores six main aspects of video content: definition of the disease, its signs and symptoms, risk factors, assessment, management, and outcomes. The scoring was divided into three main sections: no content (0 points), some content (1 point), and a lot of content (2 points).

All settings and history on a smartphone were deleted before conducting the search to avoid potential pre-buffered cache-induced targeted video recommendations, and none of the videos were downloaded, reposted, liked, or commented on to avoid impacting the data. The video evaluation process was as follows: Firstly, we recorded video information including the publication date; video duration; the number of likes, favorites, and shares; as well as publisher information including account name, self-description, and authentication status. The uploaders were classified into two main types (health professionals and non-health professionals) and identified by their account name and authentication status. The videos were also examined for whether they addressed common current *H. pylori*-related issues, such as *H. pylori* testing, whether all patients with *H. pylori* infection need to be treated, whether patients with *H. pylori* should be treated as a family unit, and the adverse reactions during *H. pylori* treatment. Finally, two independent raters assessed the content, reliability, and quality of the videos based on the DISCERN and GQS scoring systems. Before the evaluation, the two raters reviewed the official descriptions of these aforementioned tools and discussed the best way to evaluate video content using these tools, making any necessary adjustments.

## Results

### Short video characteristics

**Figure 1:**
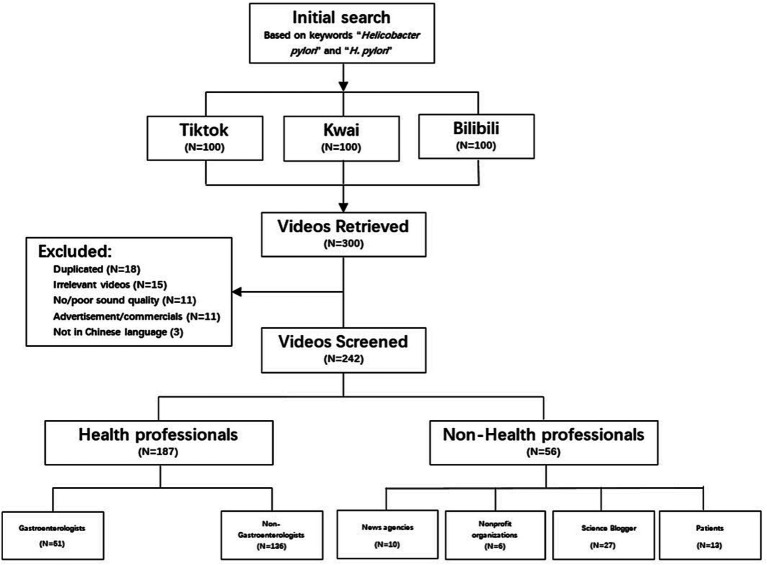
Flowchart of *H. pylori*-related videos included in the present study.

A total of 242 short videos retrieved from the three platforms were included for data analysis (Figure 1). The videos came mainly from two types of sources: health professionals and non-health professionals. In this study, most *H. pylori*-related videos were posted by health professionals (187/242, 77.3%). We further divided health professionals into gastroenterologists (51/242, 21.0%) and non-gastroenterologists (136/242, 56.2%). As for non-health professionals (55/242, 22.7), we categorized them into four types, including news agencies, nonprofit organizations, science bloggers, and patients, where science bloggers posted the most videos (26/242, 10.7%), followed by patients (13/242, 5.4%), news agencies (10/242, 4.1%), and nonprofit organizations (6/242, 2.5%) (Table 1).

**Table 1:** Characteristics of the sources of *Helicobacter pylori*–related videos (*N* = 242).

Source	Description	Videos. n (%)
		Total
*Health professionals*		
Gastroenterologists	Certified physician or nurse practitioner specializing in gastroenterology	51 (21.1)
Non-gastroenterologists	Certified physician or nurse practitioner specializing in in other medical fields except gastroenterology	136 (56.2)
*Overall*		187 (76.9)
*Non-Health professionals*		
News agencies	News agencies and the press	10 (4.1)
Nonprofit organizations	Organizations and public hospitals that operate in the collective, public or social interest	6 (2.5)
Science Blogger	Individuals engaged in the dissemination of scientific knowledge	26 (10.7)
Patients	Patients who have undergone *H. pylori* testing or treatment	13 (5.4)
*Overall*		56 (23.1)

The overall video length ranged from 92 to 2,280 s, with a median length of 198.5 s. The median number of likes received by the videos was 968 (interquartile range [IQR]: 178–5,620), and the median number of favorites and shares was 294 (IQR: 58–1,050) and 44.5 (IQR: 0–1,306), respectively (Table 2). Compared with health professionals, videos contributed by non-health professionals were longer (median: 217.5 vs. 184.5, *p* = 0.586) and shared more (median: 82 vs. 41, *p* = 0.248), but had fewer likes (median: 597 vs. 1093.5, *p* = 0.164) and favorites (median: 244.5 vs. 294, *p* = 0.225) (Supplementary Table S3). We further compared characteristics of videos contributed by gastroenterologists and non-gastroenterologists (Supplementary Table S4) and found that the median length (median: 231 vs. 182, *p* = 0.489) and number of shares (median: 43 vs. 38, *p* = 0.744) of videos provided by gastroenterologists were greater than those posted by non-gastroenterologists, but the differences were not statistically significant. In contrast, the median number of likes (median: 1320 vs. 565, *p* = 0.019) and favorites (median: 454 vs. 131, *p* = 0.007) of videos posted by non-gastroenterologists was significantly more than that of videos contributed by gastroenterologists.

**Table 2:** Characteristics of *Helicobacter pylori*–related videos.

Characteristics	*n* = 242
Video duration (seconds), median, IQR	198.5 (92–2,280)
Number of likes, median, IQR	968 (178–5,620)
Number of favorites, median, IQR	294 (58–1,050)
Number of shares, median, IQR	44.5 (0–1,306)
DISCERN score, median, IQR	2 (1–3)
GQS score, median, IQR	2 (2–3)
*Family-based H. pylori infection control and management (n, %)*	
Not mentioned	228 (94.2)
Recommend	12 (5)
Not recommended	2 (0.8)
*Treat all H. pylori-positive patients with no eradication of treatment-resistant factors (n, %)*	
Not mentioned	175 (72.3)
Recommend	51 (21.1)
Not recommended	16 (6.6)
*Adverse effects (n, %)*	
Not mentioned	203 (83.9)
Mentioned	39 (16.1)

### Short video content analysis

The content comprehensiveness of each video was evaluated according to the six main aspects and common current *H. pylori* issues described in the Methods section, and the results showed that very few videos provided extensive content about *H. pylori*. As shown in Table 3, 73.2% of the videos contained little or no content on *H. pylori* definition, and the risk factors for *H. pylori* infection were rarely or not at all addressed in 68.3% of videos. Moreover, there were few videos (13.2%) describing *H. pylori* testing methods in detail. Treatment or management of *H. pylori* infection was the most common topic, with 63.6% of videos providing information on the management of *H. pylori*.

**Table 3:** Completeness of video content.

Video content	Definition, *n* (%)	Signs/symptoms, *n* (%)	Risk factors, *n* (%)	Test, *n* (%)	Treatment/Management, *n* (%)	Outcomes, *n* (%)
No content (0 points)	164 (67.8)	134 (55.4)	158 (65.3)	152 (62.8)	84 (34.7)	109 (45)
Little content (0.5 points)	13 (5.4)	7 (2.9)	8 (3.3)	12 (5)	4 (1.7)	9 (3.7)
Some content (1 point)	36 (14.9)	50 (20.7)	45 (18.6)	46 (19)	99 (40.9)	90 (37.2)
Most content (1.5 points)	5 (2.1)	6 (2.5)	10 (4.1)	8 (3.3)	6 (2.5)	15 (6.2)
Extensive content (2 points)	24 (9.9)	45 (18.6)	21 (8.7)	24 (9.9)	49 (20.2)	19 (7.9)

**Figure 2:**
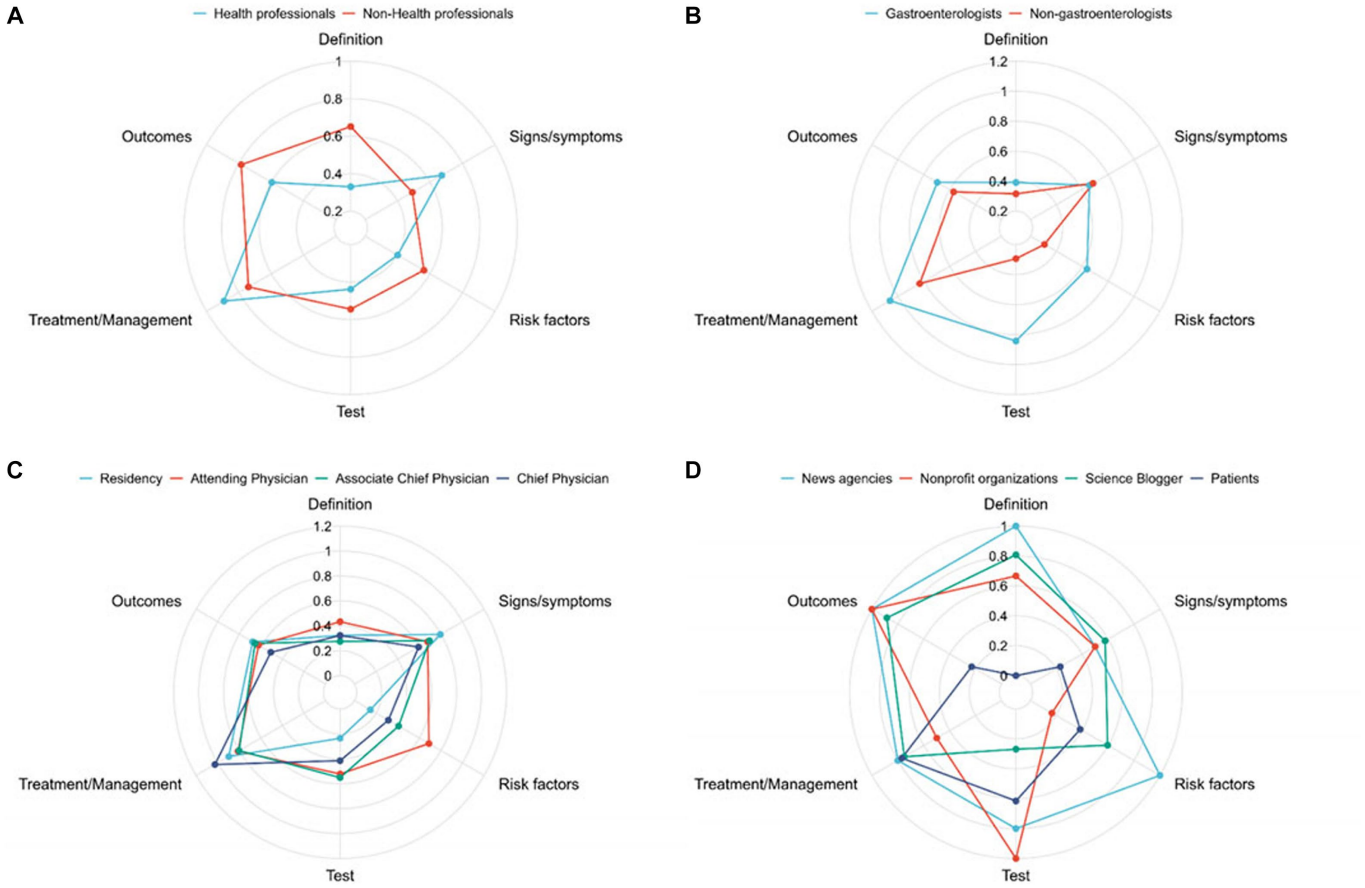
Comparisons of content comprehensiveness **(A)** between health professionals and non-health professionals; **(B)** between gastroenterologists and non-gastroenterologists; **(C)** among residents, attending physicians, associate chief physicians, and chief physicians; **(D)** among news agencies, nonprofit organizations, science bloggers, and patients.

Figure 2 shows comparisons in the video content of different subgroups. Overall, videos posted by healthcare professionals provide more information about the symptoms and treatment of *H. pylori*, while non-healthcare professionals focus on the definition, detection, outcomes, and risk factors of *H. pylori*. Among healthcare professionals, gastroenterologists’ videos have higher content coverage in all six aspects, especially in the treatment and detection of *H. pylori*. However, both types of videos lack substantial content coverage in the definition of *H. pylori*. Further subgroup analysis based on professional titles, including residents, attending physicians, associate chief physicians, and chief physicians, showed no substantial differences in the content coverage of videos related to *H. pylori* definition, symptoms, screening, and outcomes among different healthcare professionals. However, videos from attending physicians are more likely to address risk factors for *H. pylori* infection, while videos from chief physicians focus more on *H. pylori* treatment. As for non-healthcare professionals, videos from news agencies provide more comprehensive information about *H. pylori*, while videos from patients are limited in covering all six aspects of *H. pylori* information.

**Figure 3:**
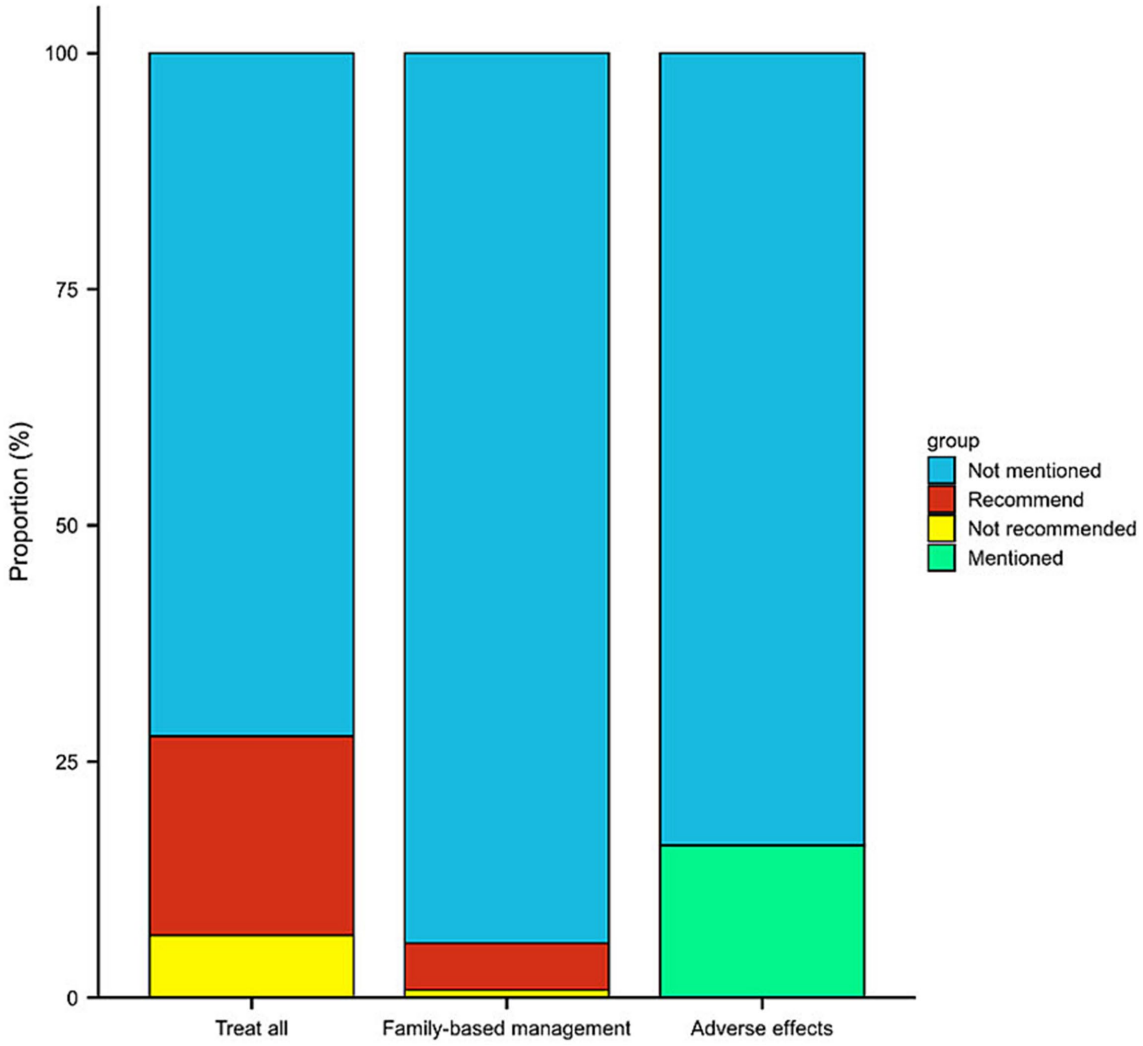
Percentage of short videos with common *H. pylori* questions (whether all positive patients should be eradicated, whether testing and treatment based on family, whether adverse effects of eradication are mentioned).

Furthermore, we assessed whether these videos addressed current topics related to *H. pylori*. As shown in Figure 3, most videos did not mention whether all *H. pylori*-positive patients need eradication treatment (175/242, 72.3%), whether *H. pylori* should be eradicated at the family level (228/242, 94.2%), and whether *H. pylori* eradication treatment has any adverse effects (203/242, 83.9%). Further subgroup analysis revealed that videos from health professionals are more likely to be associated with family-based *H. pylori* eradication (6.5% vs. 0) and the need for eradication in all positive patients (23.7% vs. 12.5%) (Supplementary Table S3). Regarding health professionals, gastroenterologists’ videos are more likely to address these three questions, as shown in Supplementary Table S4.

### Short video information assessment

**Figure 4:**
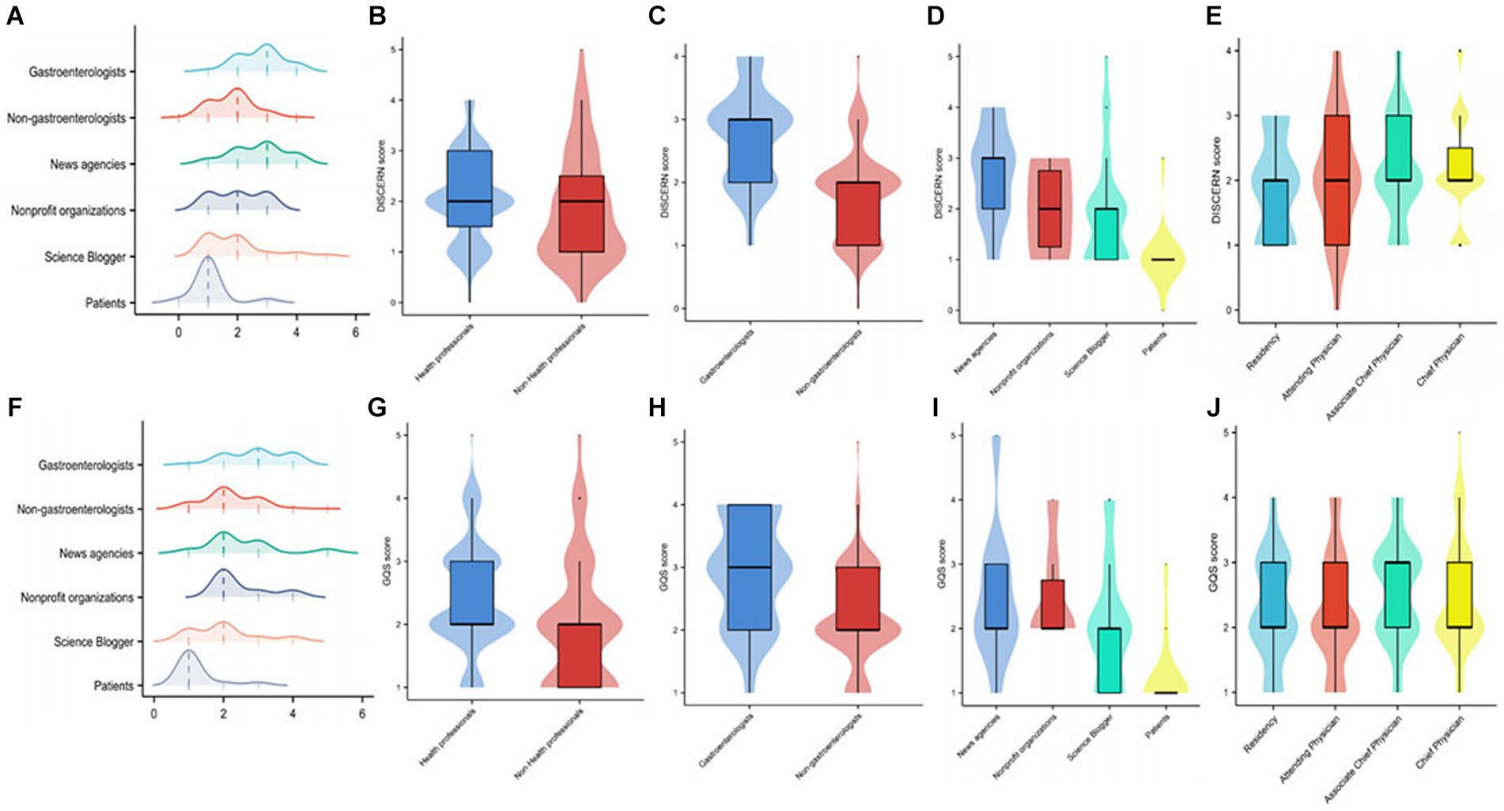
Comparison between DISCERN scores and Global Quality Scores (GQS) across video sources. **(A)** The distribution of DISCERN scores among different sources displayed by the ridge plot. **(B–E)** Comparisons of DISCERN scores in videos from different sources displayed by violin plots. **(F)** The distribution of GQS scores among different sources displayed by the ridge plot. **(G–J)** Comparisons of GQS scores in videos from different sources displayed by violin plots; *p < 0.05; **p < 0.01; ns: non-significant.

As shown in Table 2, overall video content quality was low, with a median GQS score of 3 (IQR: 2–3). There was no significant difference in video quality between health professionals and non-health professionals. Among health professionals, videos from gastroenterologists had significantly higher quality than videos from non-gastroenterologists, with no association between physician title and video quality. Among non-health professionals, news agencies posted the best quality videos. We also evaluated video reliability, which was low overall, while the subgroup analysis showed no difference between the reliability of videos posted by health and non-health professionals. Video reliability was significantly higher for gastroenterologists than for non-gastroenterologists, and physician title was not associated with video reliability. The lowest reliability videos were posted by patients among non-health professionals (Figure 4).

## Discussion

### High-quality health education is an important factor in *Helicobacter pylori* eradication

Gastric cancer has one of the highest incidence and mortality rates among gastrointestinal cancers worldwide, especially in East Asia, and *H. pylori* infection is the major risk factor associated with gastric cancer (22–24). Although significant progress has been made in understanding the relationship between *H. pylori* infection and gastric cancer, the clinical success rate of *H. pylori* eradication remains unsatisfactory (25, 26). Patient awareness of the disease is a crucial factor affecting the eradication and recurrence rate of *H. pylori* (27–29). Improved knowledge of *H. pylori*, its detection methods, infection risk factors, and management not only can reduce patient fear of *H. pylori* and help patients gain or maintain the skills they need to best manage their lives to reduce the spread of *H. pylori*, but also can increase the compliance of patients, improving the eradication rate of *H. pylori* (26). Several previous studies have shown that high-quality education about *H. pylori* is effective in improving the *H. pylori* eradication rate (26, 30, 31).

### Short video plays an important role in health education

According to the 49th Statistical Report on Internet Development in China, the number of mobile phone users in China has reached 1.643 billion, and the coverage of short videos among Chinese netizens has reached 90.5%, which means that nearly everyone in China can get or receive information through short videos on mobile phones (32). Through short videos, people can obtain health information more conveniently, and compared with traditional textual information platforms, this readily accessible platform provides people with graphical video information, which makes it easier for users to absorb and remember information (10). For example, during the coronavirus pandemic, there were 93.1 billion views of pandemic-related videos on TikTok by July 2020 (33). The results of Song et al. also showed that short health videos on TikTok had received approximately 1.7 million likes and 176,000 comments since they were published (18). The 242 short videos analyzed in this study had received approximately 6.2 million likes and were shared 1.2 million times, which suggests that short videos about *H. pylori* are widely viewed and disseminated. Therefore, health education for patients through short videos is an important tool that is effective as well as inexpensive. However, the quality of videos varies, and some videos even spread erroneous health information, which can cost patients more effort in treating *H. pylori*. Therefore, it is important for health practitioners to review the content of videos and screen the videos for high-quality information, ensuring their effectiveness in health education and disease self-management of patients.

### Principal findings

Currently, several studies have evaluated the quality of *H. pylori*-related videos on short video platforms (34–36). Ergenç et al. (34) assessed *H. pylori*-related videos on YouTube, while Tan et al. and Du et al. evaluated the quality of such videos on TikTok and Bilibili (35, 36). Although they all concluded that the quality of videos on short video platforms is unsatisfactory, their studies still suffer from limitations such as small sample sizes and a single-platform evaluation. Besides, these studies did not make further differentiations among healthcare workers when categorizing video sources. In reality, videos posted by doctors from specialized departments in disease research tend to have significantly higher quality than those from doctors in other departments (10, 37). This may be a reason for the inaccurate conclusion drawn by these studies that non-profit organization videos have higher quality than doctor-generated videos. Additionally, these studies did not address topics of significant interest to people, such as whether all *H. pylori*-positive patients need eradication and whether testing for *H. pylori* eradication should be done on a family basis. Therefore, there is still a need for a larger-sample, multi-platform, and more comprehensive study.

In this study, we evaluated the content, quality, and reliability of health education videos about *H. pylori* on TikTok, Kwai, and Bilibili. Overall, the quality of videos in our sample was poor, with few videos comprehensively addressing the six aspects of *H. pylori* infection: definition, symptoms, risk factors, detection, treatment, and outcomes, making the video content potentially less helpful to patients, which may be related to the fact that the videos were not regulated and monitored before releasing.

Overall, videos from health professionals and news media provide accurate information on antibiotic-based triple or quadruple therapy for *H. pylori* treatment. In terms of preventive measures, videos from health professionals and news media sources correctly emphasize preventive actions such as using public chopsticks and reducing dining out to prevent transmission. There was no significant difference in the quality and reliability of videos posted by health professionals and non-health professionals. A subgroup analysis of health professionals showed that non-gastroenterologists posted the greatest number of videos, which may be the reason for the low quality of videos posted by health professionals. As we watched the videos, we found that there were still many errors and biases in the understanding of *H. pylori* among non-gastroenterologists, which may be related to their different areas of expertise and lack of professional training, leading to an incomplete understanding of *H. pylori*. For example, professionals from the Department of Traditional Chinese Medicine disregarded the standard eradication protocols proposed by the global eradication guidelines and even advocated for uncertified Chinese herbal medicine for *H. pylori* eradication in the videos, resulting in misconceptions that may not only lead to the failure of *H. pylori* eradication but also affect patients’ subsequent eradication treatment. In videos posted by non-professionals, especially those from patient sources, there was an excessive exaggeration or minimization of the harm caused by *H. pylori*. Moreover, these videos even suggested incorrect treatment methods such as dietary therapy for the treatment of *H. pylori.*

We also conducted a subgroup analysis of professionals based on their titles and found no association between title and video quality. This may be because chief physicians in gastroenterology posted less video content, while chief physicians in other specialties with less knowledge about *H. pylori* posted more videos. Furthermore, most of the video content did not address current issues concerning *H. pylori.* For example, there are guidelines that *H. pylori*-positive patients need to undergo eradication therapy if there are no contraindications to eradication (3, 28, 29, 38), but most videos in this study did not mention this information, and some videos even suggested the opposite, which can create confusion about the treatment process and is not conducive for the successful eradication of *H. pylori*. Moreover, according to a previous study, *H. pylori* has a family infection rate of 71.21% in China (39), and family-based *H. pylori* testing, treatment, and guidelines are recommended for *H. pylori* eradication (3). Yet, there were essentially no videos on this aspect of *H. pylori*, which is important in improving the eradication rate and reducing the recurrence rate of *H. pylori*.

### Future directions and limitations

Short videos can make complex health information more understandable and impressive (40–42). However, incorrect health information in short videos can make self-management of disease extremely difficult. Therefore, it is important to improve the information quality of short videos to maintain patients’ health and reduce their financial burden. To this end, we propose the following recommendations to make short videos a truly important tool for health education in the future. Firstly, the relevant departments should carefully review the content of health promotion videos before they are released to prevent the dissemination of wrong information through the Internet. Secondly, we should advocate for more authoritative videos from relevant professionals, and if necessary, the government or authoritative institutions should invite chief physicians of relevant professions to give detailed and easy-to-understand explanations on self-management of diseases and answer common questions of patients. Finally, the video platforms should keep the authenticated authoritative videos on top, so that patients can view them first when searching for related content and avoid being misled by other misinformation.

In the future, videos related to *H. pylori* should provide more detailed explanations in terms of definition, symptoms, detection, treatment, risk factors, and outcomes. At the same time, content that concerns patients, such as whether all *H. pylori*-positive patients need eradication treatment, whether *H. pylori* should be eradicated at the family level, and adverse reactions after *H. pylori* treatment, should be explained in dedicated videos by authoritative gastroenterology experts and promoted through official accounts to avoid patients receiving misinformation in these areas.

There are still some limitations in this study. Firstly, only Chinese videos were included for analysis; therefore, the results of this study may not be applicable to short video platforms in other languages. Secondly, since videos are constantly uploaded and deleted, this study only evaluated and analyzed videos posted within a specific period.

Third, due to privacy concerns, we did not obtain the accurate number of views of the videos. However, based on the number of likes, shares, and bookmarks, we can still conclude that these videos have a considerable viewership on Chinese short video platforms, with a broad audience. Besides, in this study, a detailed subgroup analysis of video sources was conducted, and a relevant evaluation was made regarding whether the videos addressed common *H. pylori* issues. Thus, the findings of this study provide important guidelines for both patient selection of short videos and subsequent management of content on short video platforms.

## Conclusion

A total of 242 *H. pylori*-related short videos from three short video platforms (TikTok, Kwai, and Bilibili) were analyzed in this study. Overall, the content and information quality of the videos were not very satisfactory and video quality was correlated with the source of the videos, with the highest quality of videos posted by gastroenterologists. Although most of the videos mentioned the relationship between *H. pylori* infection and gastric cancer and advocated the need for *H. pylori* eradication, the videos did not address topics of current concern, such as whether all *H. pylori*-positive patients need to undergo eradication treatment, whether family-based testing and eradication should be performed, and the adverse effects of eradication treatment. In the future, *H. pylori*-related short videos need to be further improved to more accurately disseminate *H. pylori*-related knowledge to patients, thereby increasing the success rate of *H. pylori* eradication.

## Data availability statement

The original contributions presented in the study are included in the article/Supplementary material, further inquiries can be directed to the corresponding authors.

## Author contributions

YL: Data curation, Formal analysis, Investigation, Methodology, Project administration, Writing – original draft. FL: Conceptualization, Data curation, Investigation, Methodology, Writing – original draft. ZH: Investigation, Methodology, Project administration, Validation, Writing – original draft. WL: Formal analysis, Investigation, Project administration, Supervision, Writing – review & editing. CZ: Formal analysis, Investigation, Project administration, Resources, Writing – original draft. YD: Conceptualization, Methodology, Project administration, Supervision, Writing – review & editing. ZL: Investigation, Project administration, Supervision, Validation, Writing – review & editing.
