# Supplementary material for: The status quo of short videos as a health information source of Helicobacter pylori: a cross-sectional study
Source: Front Public Health. 2024 Jan 8;11:1344212. doi: 10.3389/fpubh.2023.1344212 (PMC10800962; doi:10.3389/fpubh.2023.1344212)
Supplement: Supplementary file 2 [file Table_2.DOCX]

**Supplementary Table 2. Global Quality Score (GQS) for assessing the reliability and quality of video. (Scoring ranges from 1 to 5)**

| **GQS Definition** | **Score** |
| --- | --- |
| Poor video quality: Specifically, the content is illogical, poorly flowing, with most information missing and useless to patients | 1 |
| Poor quality in general: the specific performance is realized as follows: poor logic of the content, although some information is listed, but still missing more important information, which is of very limited use to patients | 2 |
| Moderate quality: this is demonstrated by the fact that there is some logic and some important information is fully discussed | 3 |
| Good quality: specifically, the video is logical and smooth, with most relevant information covered and useful to patients | 4 |
| Excellent quality: specifically, the video is logical and the content is very smooth and very useful for patients | 5 |
