# Supplementary material for: The status quo of short videos as a health information source of Helicobacter pylori: a cross-sectional study
Source: Front Public Health. 2024 Jan 8;11:1344212. doi: 10.3389/fpubh.2023.1344212 (PMC10800962; doi:10.3389/fpubh.2023.1344212)
Supplement: Supplementary file 3 [file Table_3.DOCX]

| **Supplementary Table 3. Comparison of *H. pylori*-related video characteristics between health professionals and non-health professionals** | | | |
| --- | --- | --- | --- |
| **Characteristics** | **Health professionals (n=186)** | **Non-health professionals (n=56)** | **p** |
| Video duration (seconds), median, IQR | 184.5 (89-2280) | 217.5 (117-1530) | 0.586 |
| Number of likes, median, IQR | 1093.5 (238-5620) | 597 (124-5711.5) | 0.164 |
| Number of favorites, median, IQR | 294 (65-1052) | 244.5 (24.5-883.5) | 0.225 |
| Number of shares, median, IQR | 41 (0-1516) | 82 (6-1088) | 0.248 |
| DISCERN score, median, IQR | 2 (1-3) | 2 (1-3) | 0.078 |
| GQS score, median, IQR | 2 (2-3) | 2 (1-2.5) | 0.002 |
| **Family-based H. pylori infection control and management** |  |  | 0.084 |
| Not mentioned | 173 (93) | 55 (98.2) |  |
| Recommend | 12 (6.5) | 0 |  |
| Not recommended | 1 (0.5) | 1 (1.8) |  |
| **Treat all H. pylori-positive patients with no eradication of treatment-resistant factors** |  |  | 0.103 |
| Not mentioned | 132 (71) | 43 (76.8) |  |
| Recommend | 44 (23.7) | 7 (12.5) |  |
| Not recommended | 10 (5.3) | 6 (10.7) |  |
| **Adverse effects** |  |  | 0.145 |
| Not mentioned | 160 (86) | 43 (76.8) |  |
| Mentioned | 26 (14) | 13 (23.2) |  |
|  |  |  |  |
