# Supplementary material for: The status quo of short videos as a health information source of Helicobacter pylori: a cross-sectional study
Source: Front Public Health. 2024 Jan 8;11:1344212. doi: 10.3389/fpubh.2023.1344212 (PMC10800962; doi:10.3389/fpubh.2023.1344212)
Supplement: Supplementary file 4 [file Table_4.DOCX]

| **Supplementary Table 4. Comparison of *H. pylori*-related video characteristics between gastroenterologists and non-gastroenterologists** | | | |
| --- | --- | --- | --- |
| **Characteristics** | **Gastroenterologists (n=51)** | **Non-gastroenterologists (n=136)** | **p** |
| Video duration (seconds), median, IQR | 231 (95-2280) | 182 (89-2280) | 0.489 |
| Number of likes, median, IQR | 565 (104-3869) | 1320 (294-6268) | 0.019 |
| Number of favorites, median, IQR | 131 (28-501) | 454 (90-1124) | 0.007 |
| Number of shares, median, IQR | 43 (0-487) | 38 (0-2192) | 0.744 |
| DISCERN score, median, IQR | 3 (2-3) | 2 (1-2) | 0.001 |
| GQS score, median, IQR | 3 (2-4) | 2 (2-3) | 0.001 |
| **Family-based H. pylori infection control and management** |  |  | 0.039 |
| Not mentioned | 44 (86.2) | 130 (95.6) |  |
| Recommend | 6 (11.8) | 6 (4.4) |  |
| Not recommended | 1 (2) | 0 |  |
| **Treat all H. pylori-positive patients with no eradication of treatment-resistant factors** |  |  | 0.032 |
| Not mentioned | 34 (66.7) | 98 (72.1) |  |
| Recommend | 17 (33.3) | 28 (20.6) |  |
| Not recommended | 0 | 10 (7.3) |  |
| **Adverse effects** |  |  | 0.159 |
| Not mentioned | 41 (80.4) | 119 (87.5) |  |
| Mentioned | 10 (19.6) | 17 (12.5) |  |
